# Rush Medical College

**Published:** 1844-12

**Authors:** J. H. Bird


### ILLINOIS

### MEDICAL & SURGICAL JOURNAL.

VOL. I. DECEMBER, 1844. NO. 8. 9

### RUSH MEDICAL COLLEGE.

### PROF. BRAINARD'S CLINIC. NOV. 4, 1844.

[Reported for the Journal, by J. H. Bird.]

Case 1. —— Dayton, ætas 2½, was presented to the class, having situated upon the right side of the abdomen, a tumor, extending from the anterior superior process of the ilium upwards, four inches; and from the external margin of the rectus abdominis, six inches outwards. The skin covering and surrounding the tumor was of a livid hue, and interspersed upon its surface, were several small light spots, bearing the appearance of cicatrices. The discoloration, which was a superficial congenital mark, extended downward upon the thigh and upon the back, and was covered with hair. The tumor was hard around its base, but was more yielding to pressure at its apex, giving the signs of fluctuation. The history of the case, as learned from the parents, was, that at the period of birth, the nævus-like apperance of the skin existed, and that shortly after, a tumor of the size of a pea, was discovered in the iliac region, quite firm to the touch; that it had gradually enlarged; but that in the last six months it had advanced quite rapidly, alarming them by its progress and enormous size; the child had complained at times, of pain, but otherwise was quite healthy. The parents were of good constitution, and were also healthy.

*Remarks.*—Gentlemen, you have presented before you an encephaloid tumor: encephaloid tumors are one of the varieties of the cancerous or malignant tumors. Cancerous tumors are of three varieties, schirrus, encephaloid or cephaloma, and colloid. The schirrus, the first variety mentioned; preferring rather for its develepement, the female breast, the lips, the testicle, or it may exist in any other part of the body; presents itself as a hard and indolent tumor of small size, giving but little uneasiness to the patient, with but occasional lancinating pains. On examination, you find a hard and dense tissue crossed by fibrous bands; if you cut it, it gives a creaking sound under the scalpel, it presents very much the appearance of a turnip when cut by the knife; a schirrus tumor may remain indolent for a long time, sometimes through life, but generally sooner or later, it assumes great activity, enlarging, approaching nearer the surface, the skin over it, becoming thinner by the distension, bursts, giving issue to a sanious fluid, and the cancerous ulcer is developed.

As I shall speak more in detail on the subject of schirrus in its proper place in the course, I now merely will make a few remarks on the variety before us. The encephaloid is called so, from the resemblance of its substance to cerebral matter. This variety may appear in all parts of the body and also may exist at the same time with the schirrus. In its early development, it is not as dense or firm as schirrus, but is much more active in its progress; on examination you find it cellular in its structure, occasioned by the passage of fibrous bands across it. In its progress, the substance contained within the cells, becomes softened, appearing quite similar to the substance of the brain in infants; and finally like schirrus, it bursts through the skin and the ulcer appears; its appearance, &c., I defer until I reach it in the course. The removal of the tumor, which is the only remedy, often hastens the progress of the disease; still in certain cases, of which this is an instance, an operation would be advisable; about which we will proceed. I shall, after making an incision, dissect out all the morbid tissue and endeavor to save enough of the skin to induce its healing by the first intention.

The Doctor commenced by making a transverse incision across, and then dissected out the tumor, a part of the superficial abdominal fascia and external oblique muscle being found diseased, were also removed; on the dissection of the lower part of the tumor, the skin was found diseased, and was also removed; the flaps of the wound were approximated by stiches and straps of adhesive plaster, and light bandages were applied to exclude the air and support the abdomen. The child bore the operation quite well, lost but little blood, no vessel requiring ligature, and appeared to evince but little exhaustion at its completion.

Nov. 5. The child is quite lively today and suffers but little uneasiness from the wound.

Nov. 11. The wound has been gradually healing to this date; the major part united by the first intention, the remainder sufficiently healed to allow of the dismissal of the patient. The examination of the tumor confirmed the diagnosis, it presented a mass of substance the greater part of which was quite softened, the fibrous bands crossing it not firm, having the appearance of cerebral matter, from which a serous fluid could be expressed; the remainder resembled more the schinus, but not so dense.

---

Case 2.—Mr. H——, ætas 33, presented himself before us for relief. Dr. Brainard remarked, many of you may recollect this patient, who presented himself before you last winter; you may recollect the starch bandage was applied, and you can observe the good effects of the application. This patient has been afflicted with white swelling of the ancle joint and caices of the inferior end of the tibia with abscess, of five years standing. He has used various remedies and amputation has been advised; and, Gentlemen, you will hardly find two practitioners agreeing as to the treatment of this disease, or in that of sprains and the like; some treating as inflammatory, others stimulating: the cold *douche* has been successfully used in cases of synovial inflammations. In the present case, I applied the immoveable apparatus or starch bandage, to which the patient ascribes his great relief; when he last presented himself before us, he was obliged to use crutches, but now he gets along quite well with two sticks. The Dr. explained the mode of preparation of the paste bandage; he spoke of the good effects resulting from its application in similar cases and in cases of fracture, &c. After the application of the bandage, the patient was requested to report himself at times, that we might judge of its effects. Dr. B. remarked that the bandage would be applied for six months and a cure might be expected, and that notwithstanding the immobility of the articulation, the patient might be able to plod his way through life without much inconvenience.

---

Case 3.—Mr. W. having a fracture of the melacarpal bone of the forefinger, presented himself. Remarks—I was not called at the time of the accident producing the fracture, but it was very well dressed by another, and as the man wishes to leave town as soon as possible, I will apply the proper bandages. In cases of this kind of fracture, there is not a great displacement of the bones; the effect is, an inward projection, which we will remedy by placing in the hand of the patient, a roller of suitable size, that firm pressure by it, may be made upon the fractured extremeties. The proper bandages were then applied.

---

Case 4.—Miss D. having a polypus in the nose, applied for relief. Dr. Brainard remarked: Gentlemen, if I should speak of all the diseases to which the nasal membrane is subject, I would enter into the history of nearly all the specific diseases. This membrane is liable to inflammation and its various effects, as in catarrh; also to the formation of various morbid products. The polypus of the nose has several varities; the gelatinous, the vascular, fleshy, and several other kinds; they arise from the mucous membrane and generally in the middle meatus. In examining the structure of the nose, you may observe three channels or meatus's communicating with the pharyux; the superior, the middle formed by the two turbinated bones, the inferior, by the inferior turbinated bone and the floor of the nostril. The gelatinous polypus is a jelly-like substance, of a pear shape, attached by a small pedicle; the vascular bleeds at the touch. In the progress of the polypus, it presses upon all the surrounding parts, invading the opposite nostril, upon the orbit, and has even encroached upon the bones of the palatine arch. The patient feels as if he had a cold in the head, and the action of respiration impeded; each inspiration or expiration affecting the situation of the polypus. The best mode of cure, is by tearing the polypus from its attachments, by means of forceps; the force used must be gradual, to prevent the tearing off of a part: in cases of hemorrhage resulting from the partial removal, the removal of the remainder is the indication. In this case, our patient first perceived the polypus, three years ago, it has been gradually increasing in size, giving her much inconvenience, and she now desires its removal. On examination I find it to be gelatinous, and I hope to remove it without much difficulty. The Dr. then tore it from its situation and also a piece of the middle turbinated bone, to which it attached; very little hemorrhage resulted from the operation. Dr. B. then remarked, I will dismiss the patient for the present, and if on future examination, I may find other similar growths, which I think not the case, the proper means will be pursued for their removal. The next day the nostril was perfectly free and the patient returned home.

---

Case 5.—Mr. ——— having a cutaneous cancer upon the arm, applied for its removal. Dr. B. remarked: Gentlemen, this man has a schirrus tumor of small size upon the arm, and wishes it to be removed without cutting. We have the advantage over empirics, inasmuch as we may effect our purpose in two ways, by the knife and by caustic. Patients dread the use of the knife, as even do surgeons, upon themselves, still the caustic is more painful. We will apply the solid caustic potash or potassa fusa to the surface, and you can judge of its effect; the caustic potash is quite deliquescent, therefore you apply it upon a less surface than is to be affected: a very good preparation for similar purpose, is a combination of the potash with quick lime, formed into a paste with alcohol; this form is good to prevent the deliquescence of the potash, and its irritating the surrounding surface. The caustic was applied till a profound eschar was produced. Dr. B. remarked that the after treatment in this case would be a few dressings of simple cerate, allowing of its cicatrization.

